# Bis(carboxy­meth­yl)ammonium 4-toluene­sulfonate

**DOI:** 10.1107/S1600536808026214

**Published:** 2008-08-20

**Authors:** Kong Mun Lo, Seik Weng Ng

**Affiliations:** a Department of Chemistry, University of Malaya, 50603 Kuala Lumpur, Malaysia

## Abstract

The imino­diacetic acid component of the title salt, C_4_H_8_NO_4_^+^·C_7_H_7_SO_3_^−^, is protonated at the N atom. The cation uses the ammonium group to form hydrogen bonds to the O atoms of two adjacent sulfonate groups. In addition, the carboxylic acid portions of the cation form hydrogen bonds to the sulfonate groups. The hydrogen-bonding inter­actions give rise to a layer structure.

## Related literature

For the crystal structures of imino­diacetic acid hydro­halides, see: Oskarsson (1973 ▶, 1974*a* ▶,*b* ▶, 1976 ▶).
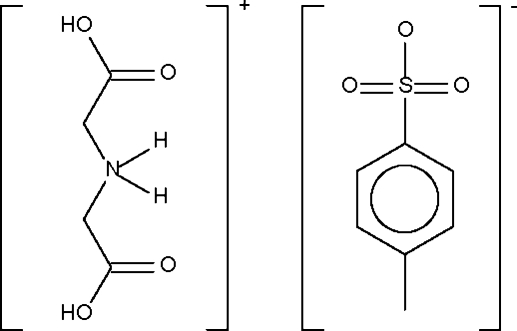


## Experimental

### 

#### Crystal data

C_4_H_8_NO_4_^+^·C_7_H_7_O_3_S^−^
*M* *_r_* = 305.30
Orthorhombic, 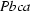

*a* = 9.9291 (2) Å
*b* = 10.3636 (2) Å
*c* = 25.8862 (5) Å
*V* = 2663.72 (9) Å^3^
*Z* = 8
Mo *K*α radiation
μ = 0.28 mm^−1^
*T* = 100 (2) K
0.27 × 0.27 × 0.27 mm

#### Data collection

Bruker SMART APEX diffractometer
Absorption correction: multi-scan (*SADABS*; Sheldrick, 1996 ▶) *T*_min_ = 0.929, *T*_max_ = 0.929
20842 measured reflections
3059 independent reflections
2560 reflections with *I* > 2σ(*I*)
*R*_int_ = 0.048

#### Refinement

*R*[*F* ^2^ > 2σ(*F* ^2^)] = 0.038
*wR*(*F* ^2^) = 0.119
*S* = 1.15
3059 reflections
198 parameters
4 restraints
H atoms treated by a mixture of independent and constrained refinement
Δρ_max_ = 0.42 e Å^−3^
Δρ_min_ = −0.50 e Å^−3^

### 

Data collection: *APEX2* (Bruker, 2007 ▶); cell refinement: *SAINT* (Bruker, 2007 ▶); data reduction: *SAINT*); program(s) used to solve structure: *SHELXS97* (Sheldrick, 2008 ▶); program(s) used to refine structure: *SHELXL97* (Sheldrick, 2008 ▶); molecular graphics: *X-SEED* (Barbour, 2001 ▶); software used to prepare material for publication: *publCIF* (Westrip, 2008 ▶).

## Supplementary Material

Crystal structure: contains datablocks global, I. DOI: 10.1107/S1600536808026214/bh2187sup1.cif

Structure factors: contains datablocks I. DOI: 10.1107/S1600536808026214/bh2187Isup2.hkl

Additional supplementary materials:  crystallographic information; 3D view; checkCIF report

## Figures and Tables

**Table 1:** Hydrogen-bond geometry (Å, °)

*D*—H⋯*A*	*D*—H	H⋯*A*	*D*⋯*A*	*D*—H⋯*A*
N1—H1*N*1⋯O2^i^	0.88 (1)	2.02 (1)	2.885 (2)	167 (2)
N1—H1*N*2⋯O3^ii^	0.88 (1)	2.06 (2)	2.792 (2)	140 (2)
O5—H5*O*⋯O1	0.84 (1)	1.79 (1)	2.607 (2)	164 (3)
O7—H7*O*⋯O2^iii^	0.84 (1)	1.85 (1)	2.659 (2)	160 (3)

Symmetry codes: (i) 
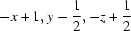
; (ii) 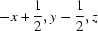; (iii) 
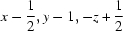
.

**Fig. 1.**
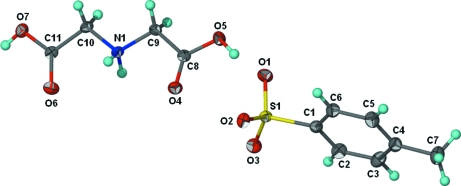
Thermal ellipsoid plot (Barbour, 2001) of (C4H8NO4)+(C7H7O3S)- at the 70% probability level. Hydrogen atoms are drawn as spheres of arbitrary radius.

## supplementary crystallographic information

### Comment

(type here to add)

### Experimental

Iminodiacetic acid (0.55 g, 4 mmol) and *p*-toluenesulfonic acid (0.65 g, 4 mmol) were heated in toluene (100 ml) for 1 h. Crystals were isolated from the cool solution after several days.

### Refinement

Carbon-bound H-atoms were placed in calculated positions (C—H 0.95 to 0.99 Å) and were included in the refinement in the riding model approximation, with *U*_iso_(H) set to 1.2 to 1.5*U*_eq_(carrier C). The acid and ammonium H atoms were refined with distance restraints of O—H = 0.84 (1) and N—H = 0.88 (1) Å; their isotropic displacement parameters were freely refined.

### Crystal data

**Table:**

C_4_H_8_NO_4_^+^·C_7_H_7_O_3_S^–^	*F*_000_ = 1280
*M**_r_* = 305.30	*D*_x_ = 1.523 Mg m^−^^3^
Orthorhombic, *P**b**c**a*	Mo *K*α radiation λ = 0.71073 Å
Hall symbol: -P 2ac 2ab	Cell parameters from 4293 reflections
*a* = 9.9291 (2) Å	θ = 2.9–27.2º
*b* = 10.3636 (2) Å	µ = 0.28 mm^−^^1^
*c* = 25.8862 (5) Å	*T* = 100 (2) K
*V* = 2663.72 (9) Å^3^	Triangular block, colorless
*Z* = 8	0.27 × 0.27 × 0.27 mm

### Data collection

**Table:**

Bruker SMART APEX diffractometer	3059 independent reflections
Radiation source: fine-focus sealed tube	2560 reflections with *I* > 2σ(*I*)
Monochromator: graphite	*R*_int_ = 0.048
*T* = 100(2) K	θ_max_ = 27.5º
ω scans	θ_min_ = 1.6º
Absorption correction: multi-scan(SADABS; Sheldrick, 1996)	*h* = −12→7
*T*_min_ = 0.930, *T*_max_ = 0.930	*k* = −13→13
20842 measured reflections	*l* = −33→33

### Refinement

**Table:**

Refinement on *F*^2^	Secondary atom site location: difference Fourier map
Least-squares matrix: full	Hydrogen site location: inferred from neighbouring sites
*R*[*F*^2^ > 2σ(*F*^2^)] = 0.038	H atoms treated by a mixture of independent and constrained refinement
*wR*(*F*^2^) = 0.119	*w* = 1/[σ^2^(*F*_o_^2^) + (0.0644*P*)^2^ + 0.8206*P*] where *P* = (*F*_o_^2^ + 2*F*_c_^2^)/3
*S* = 1.15	(Δ/σ)_max_ = 0.001
3059 reflections	Δρ_max_ = 0.42 e Å^−^^3^
198 parameters	Δρ_min_ = −0.50 e Å^−^^3^
4 restraints	Extinction correction: none
Primary atom site location: structure-invariant direct methods	

### Fractional atomic coordinates and isotropic or equivalent isotropic displacement parameters (Å^2^)

**Table:**

	*x*	*y*	*z*	*U*_iso_*/*U*_eq_	
S1	0.49041 (5)	0.84948 (4)	0.358654 (17)	0.01248 (14)	
O1	0.55419 (15)	0.74172 (13)	0.38508 (5)	0.0195 (3)	
O2	0.56209 (14)	0.88382 (13)	0.31102 (5)	0.0163 (3)	
O3	0.34727 (14)	0.83294 (13)	0.35029 (5)	0.0190 (3)	
O4	0.40223 (14)	0.52637 (13)	0.30853 (5)	0.0171 (3)	
O5	0.51261 (16)	0.49339 (13)	0.38301 (5)	0.0199 (3)	
H5O	0.510 (3)	0.5747 (10)	0.3841 (10)	0.034 (7)*	
O6	0.20627 (14)	0.17528 (13)	0.21695 (5)	0.0176 (3)	
O7	0.29656 (14)	−0.02406 (13)	0.22217 (5)	0.0163 (3)	
H7O	0.229 (2)	−0.047 (3)	0.2048 (10)	0.051 (9)*	
N1	0.37466 (17)	0.27383 (15)	0.28845 (6)	0.0129 (3)	
H1N1	0.402 (3)	0.316 (2)	0.2610 (7)	0.031 (7)*	
H1N2	0.2899 (11)	0.296 (2)	0.2924 (8)	0.014 (5)*	
C1	0.50864 (19)	0.98391 (18)	0.40013 (7)	0.0141 (4)	
C2	0.3995 (2)	1.02980 (19)	0.42797 (8)	0.0187 (4)	
H2	0.3146	0.9879	0.4256	0.022*	
C3	0.4152 (2)	1.1376 (2)	0.45941 (8)	0.0212 (4)	
H3	0.3404	1.1681	0.4788	0.025*	
C4	0.5374 (2)	1.20164 (19)	0.46306 (7)	0.0183 (4)	
C5	0.6471 (2)	1.15329 (19)	0.43556 (8)	0.0191 (4)	
H5	0.7319	1.1952	0.4380	0.023*	
C6	0.6335 (2)	1.04440 (19)	0.40462 (7)	0.0171 (4)	
H6	0.7093	1.0112	0.3866	0.021*	
C7	0.5517 (2)	1.3217 (2)	0.49545 (8)	0.0247 (5)	
H7A	0.4636	1.3631	0.4993	0.037*	
H7B	0.6142	1.3815	0.4785	0.037*	
H7C	0.5868	1.2985	0.5296	0.037*	
C8	0.45172 (19)	0.45546 (18)	0.34029 (7)	0.0137 (4)	
C9	0.45326 (19)	0.31071 (18)	0.33494 (7)	0.0137 (4)	
H9A	0.5472	0.2798	0.3315	0.016*	
H9B	0.4132	0.2705	0.3660	0.016*	
C10	0.3811 (2)	0.13288 (17)	0.27813 (7)	0.0142 (4)	
H10A	0.3570	0.0845	0.3098	0.017*	
H10B	0.4738	0.1085	0.2680	0.017*	
C11	0.28461 (19)	0.09925 (18)	0.23534 (7)	0.0132 (4)	

### Atomic displacement parameters (Å^2^)

**Table:**

	*U*^11^	*U*^22^	*U*^33^	*U*^12^	*U*^13^	*U*^23^
S1	0.0104 (2)	0.0118 (2)	0.0153 (2)	−0.00084 (17)	−0.00072 (16)	0.00057 (16)
O1	0.0240 (8)	0.0125 (7)	0.0221 (7)	−0.0013 (6)	−0.0072 (6)	0.0031 (5)
O2	0.0164 (7)	0.0158 (7)	0.0168 (7)	−0.0021 (6)	0.0028 (5)	−0.0016 (5)
O3	0.0109 (7)	0.0226 (8)	0.0233 (7)	−0.0016 (6)	−0.0006 (6)	−0.0024 (5)
O4	0.0169 (7)	0.0148 (7)	0.0197 (7)	0.0007 (6)	−0.0025 (5)	0.0007 (5)
O5	0.0293 (9)	0.0111 (7)	0.0192 (7)	−0.0007 (6)	−0.0092 (6)	−0.0014 (5)
O6	0.0156 (7)	0.0155 (7)	0.0217 (7)	−0.0016 (6)	−0.0030 (5)	0.0033 (5)
O7	0.0134 (7)	0.0143 (7)	0.0211 (7)	−0.0023 (6)	−0.0006 (6)	−0.0043 (5)
N1	0.0123 (8)	0.0109 (8)	0.0154 (8)	0.0011 (6)	−0.0002 (6)	0.0000 (6)
C1	0.0144 (9)	0.0131 (9)	0.0149 (8)	−0.0001 (7)	−0.0003 (7)	0.0010 (7)
C2	0.0148 (10)	0.0198 (10)	0.0215 (9)	−0.0019 (8)	0.0032 (8)	0.0004 (8)
C3	0.0190 (11)	0.0217 (10)	0.0228 (10)	0.0025 (8)	0.0066 (8)	−0.0020 (8)
C4	0.0237 (11)	0.0162 (10)	0.0150 (9)	0.0007 (8)	−0.0001 (8)	0.0004 (7)
C5	0.0145 (10)	0.0225 (10)	0.0202 (9)	−0.0045 (8)	0.0002 (8)	−0.0023 (8)
C6	0.0130 (9)	0.0199 (10)	0.0183 (9)	0.0004 (8)	0.0014 (7)	−0.0027 (7)
C7	0.0276 (12)	0.0219 (11)	0.0247 (10)	−0.0011 (9)	0.0010 (9)	−0.0067 (8)
C8	0.0102 (9)	0.0150 (9)	0.0161 (8)	−0.0005 (7)	0.0005 (7)	0.0004 (7)
C9	0.0125 (9)	0.0133 (9)	0.0152 (9)	0.0002 (7)	−0.0026 (7)	0.0000 (7)
C10	0.0132 (9)	0.0095 (8)	0.0200 (9)	0.0004 (7)	−0.0017 (7)	−0.0009 (7)
C11	0.0108 (9)	0.0132 (9)	0.0157 (8)	−0.0019 (7)	0.0028 (7)	0.0012 (7)

### Geometric parameters (Å, °)

**Table:**

S1—O3	1.4478 (14)	C2—H2	0.9500
S1—O1	1.4547 (14)	C3—C4	1.386 (3)
S1—O2	1.4675 (13)	C3—H3	0.9500
S1—C1	1.768 (2)	C4—C5	1.394 (3)
O4—C8	1.207 (2)	C4—C7	1.507 (3)
O5—C8	1.320 (2)	C5—C6	1.390 (3)
O5—H5o	0.84 (1)	C5—H5	0.9500
O6—C11	1.205 (2)	C6—H6	0.9500
O7—C11	1.328 (2)	C7—H7A	0.9800
O7—H7o	0.84 (1)	C7—H7B	0.9800
N1—C9	1.484 (2)	C7—H7C	0.9800
N1—C10	1.486 (2)	C8—C9	1.507 (3)
N1—H1n1	0.88 (1)	C9—H9A	0.9900
N1—H1n2	0.88 (1)	C9—H9B	0.9900
C1—C2	1.385 (3)	C10—C11	1.505 (3)
C1—C6	1.394 (3)	C10—H10A	0.9900
C2—C3	1.391 (3)	C10—H10B	0.9900
			
O3—S1—O1	114.00 (9)	C4—C5—H5	119.7
O3—S1—O2	112.28 (8)	C5—C6—C1	119.93 (18)
O1—S1—O2	111.72 (8)	C5—C6—H6	120.0
O3—S1—C1	106.53 (9)	C1—C6—H6	120.0
O1—S1—C1	105.95 (9)	C4—C7—H7A	109.5
O2—S1—C1	105.63 (8)	C4—C7—H7B	109.5
C8—O5—H5O	108.2 (18)	H7A—C7—H7B	109.5
C11—O7—H7O	110 (2)	C4—C7—H7C	109.5
C9—N1—C10	112.10 (14)	H7A—C7—H7C	109.5
C9—N1—H1N1	111.7 (17)	H7B—C7—H7C	109.5
C10—N1—H1N1	109.4 (16)	O4—C8—O5	125.13 (18)
C9—N1—H1N2	110.2 (14)	O4—C8—C9	123.20 (17)
C10—N1—H1N2	108.3 (15)	O5—C8—C9	111.66 (15)
H1N1—N1—H1N2	105 (2)	N1—C9—C8	109.01 (15)
C2—C1—C6	119.85 (18)	N1—C9—H9A	109.9
C2—C1—S1	120.41 (15)	C8—C9—H9A	109.9
C6—C1—S1	119.73 (15)	N1—C9—H9B	109.9
C1—C2—C3	119.53 (19)	C8—C9—H9B	109.9
C1—C2—H2	120.2	H9A—C9—H9B	108.3
C3—C2—H2	120.2	N1—C10—C11	109.43 (15)
C4—C3—C2	121.46 (18)	N1—C10—H10A	109.8
C4—C3—H3	119.3	C11—C10—H10A	109.8
C2—C3—H3	119.3	N1—C10—H10B	109.8
C3—C4—C5	118.49 (18)	C11—C10—H10B	109.8
C3—C4—C7	121.06 (19)	H10A—C10—H10B	108.2
C5—C4—C7	120.4 (2)	O6—C11—O7	125.83 (17)
C6—C5—C4	120.68 (19)	O6—C11—C10	123.36 (17)
C6—C5—H5	119.7	O7—C11—C10	110.79 (16)
			
O3—S1—C1—C2	16.35 (19)	C3—C4—C5—C6	0.9 (3)
O1—S1—C1—C2	−105.40 (17)	C7—C4—C5—C6	−178.42 (18)
O2—S1—C1—C2	135.94 (16)	C4—C5—C6—C1	1.2 (3)
O3—S1—C1—C6	−163.36 (15)	C2—C1—C6—C5	−2.3 (3)
O1—S1—C1—C6	74.89 (17)	S1—C1—C6—C5	177.39 (15)
O2—S1—C1—C6	−43.77 (18)	C10—N1—C9—C8	175.26 (15)
C6—C1—C2—C3	1.3 (3)	O4—C8—C9—N1	−5.4 (3)
S1—C1—C2—C3	−178.42 (15)	O5—C8—C9—N1	175.76 (15)
C1—C2—C3—C4	0.9 (3)	C9—N1—C10—C11	172.17 (15)
C2—C3—C4—C5	−1.9 (3)	N1—C10—C11—O6	−6.8 (3)
C2—C3—C4—C7	177.37 (19)	N1—C10—C11—O7	174.56 (14)

### Hydrogen-bond geometry (Å, °)

**Table:**

*D*—H···*A*	*D*—H	H···*A*	*D*···*A*	*D*—H···*A*
N1—H1N1···O2^i^	0.88 (1)	2.02 (1)	2.885 (2)	167 (2)
N1—H1N2···O3^ii^	0.88 (1)	2.06 (2)	2.792 (2)	140 (2)
O5—H5O···O1	0.84 (1)	1.79 (1)	2.607 (2)	164 (3)
O7—H7O···O2^iii^	0.84 (1)	1.85 (1)	2.659 (2)	160 (3)

Symmetry codes: (i) −*x*+1, *y*−1/2, −*z*+1/2; (ii) −*x*+1/2, *y*−1/2, *z*; (iii) *x*−1/2, *y*−1, −*z*+1/2.
